# Arterial hypertension due to fructose ingestion: model based on intermittent osmotic fluid trapping in the small bowel

**DOI:** 10.1186/1742-4682-7-27

**Published:** 2010-06-25

**Authors:** Sven Kurbel

**Affiliations:** 1 Osijek Medical Faculty Dept. of Physiology J Huttlera 4, 31000 Osijek Croatia

## Abstract

Based on recently reported data that fructose ingestion is linked to arterial hypertension, a model of regulatory loops involving the colon role in maintenance of fluid and sodium homeostasis is proposed.

In normal digestion of hyperosmolar fluids, also in cases of postprandial hypotension and in patients having the "dumping" syndrome after gastric surgery, any hyperosmolar intestinal content is diluted by water taken from circulation and being trapped in the bowel until reabsorption. High fructose corn sirup (HFCS) soft drinks are among common hyperosmolar drinks. Fructose is slowly absorbed through passive carrier-mediated facilitated diffusion, along the entire small bowel, thus preventing absorption of the trapped water for several hours.

Here presented interpretation is that ingestion of hyperosmolar HFCS drinks due to a transient fluid shift into the small bowel increases renin secretion and sympathetic activity, leading to rise in ADH and aldosterone secretions. Their actions spare water and sodium in the large bowel and kidneys. Alteration of colon absorption due to hormone exposure depends on cell renewal and takes days to develop, so the momentary capacity of sodium absorption in the colon depends on the average aldosterone and ADH exposure during few previous days. This inertia in modulation of the colon function can make an individual that often takes HFCS drinks prone to sodium retention, until a new balance is reached with an expanded ECF pool and arterial hypertension. In individuals with impaired fructose absorption, even a higher risk of arterial hypertension can be expected.

## Introduction

Despite wide range of daily salt and water ingestion, sodium and fluid homeostasis is maintained through orchestrated action of aldosterone, ADH, ANP and other humoral mediators. Actions of angiotensin II and aldosterone include vasoconstriction; increased glomerular filtration with sodium reabsorption and potassium secretion in the distal tubule; increased thirst and ADH secretion [1]. Beside that, a proabsorptive trophic effect of aldosterone on the pericryptal sheath in colonic mucosa was reported [1-3] and aldosterone exposure leads to increased pericryptal sodium concentrations in the colon. ADH may be another potential mediator of colonic absorption [4]. Data that link angiotensin, aldosterone and ADH to the sodium absorption in the colon are probably related to the increased large bowel wall thickness found in chronic heart failure patients [5]. Taken together, it seems that the role of colon in sodium and fluid homeostasis is often underestimated.

The whole picture has recently become more complex when fructose ingestion was reported as an independent dietary risk factor of arterial hypertension [6]. It has been known in animal models that different carbohydrate-rich diets lead to arterial hypertension [7,8], but these data were not considered applicable to healthy individuals, or to our patients. Jalal DI et al. described association between fructose intake and blood pressure levels in 4528 adults from the National Health and Nutrition Examination Survey. The cross-sectional association between the fructose intake as added sugar and blood pressure was examined in 4528 adults 18 years of age or older with no history of hypertension. Estimation of the fructose intake included fruit juices, soft drinks, bakery products and candy. Multivariate logistic regression analysis showed that high fructose intake is independently associated with higher blood pressure levels (median fructose intake was 74 grams/day, or 2.5 sugary soft drinks a day). This intake was associated with an increased odds of blood pressure 140/90 mmHg of 1.33 (95% confidence intervals 1.09 to 1.62, p = 0.005). Beside that, even after adjusting for demographics, comorbidities, physical activity, total kilocalorie, salt and vitamin C intakes, the same fructose intake leads to a 28%, 36% and 87% higher risk for arterial blood pressures above 135/85, 140/90, and 160/100 mmHg respectively [6]. These results clearly suggest that despite obesity and dietary salt, the high fructose intake in the Western diet might become an independent risk factor of developing arterial hypertension in previously normotensive adults.

In other words, although obesity *per se *is closely linked to hypertension, data reported by Jalal et al. do not suggest that obesity play an intermediary role in the reported hypertension - HFCS consumption link.

This theoretic paper is an attempt to interpret the reported link of fructose ingestion and arterial hypertension by proposing a model of increased sodium absorption in the large bowel, due to stimulation of aldosterone secretion by osmotic loads in the upper digestive tract.

## Osmotic loads of common fluids in our diet

Many isotonic "sport" drinks contain low amounts of carbohydrate (near 6%) and electrolytes [9]. Various combinations of monosaccharides and sucrose are often used to obtain isotonicity. If we look at other frequently ingested fluids, their osmolarity range is very wide. Reported data on commercial soups [9] show that the calculated osmolarity from their salt content (8.9 - 12.7 g/L) ranges from 300 to 435 mosm/L.

High fructose corn syrup (HFCS) is produced by splitting starch into glucose molecules that are partially enzymatically converted to fructose. Typical HFCS for soft drinks contains 55% fructose and 45% glucose. In comparison to pure sucrose solutions, the HFCS soft drinks contain twice as many osmotically active molecules for the same amount of sugar. Reported sugar content data of non diet soft drinks [9-11] range from 100 to 125 g/L. If only sucrose (molar mass 342.3 g/mol) is used in these beverages, the expected calculated median of sugar generated osmolarity would be from 292 to 365 mosm/L, resulting in mildly hypertonic sucrose solution. If the same amount of sugar (100 to 125 g/L) is added as pure monosaccharides (i.e., by adding HFCS, molar mass 180.16 g/mol), the calculated osmolarity would be doubled, from 555 to 694 mosm/L, in concordance with reported data on the soft drink osmolarity [9,11].

Wines and beers have even higher osmotic values [9], mainly due to their ethanol content. Even weak ethanol solution of only 4%vol has 950 mosm/L [12]. Overall osmolarity of regular beers (ethanol and other solutes, mainly carbohydrates) ranges from 1050 to 1750 mosm/L, while wines and other alcohol beverages reach even higher values [9,12].

## Intestinal fluid traffic following ingestion of hyperosmolar liquid

Similar to kitchen salt, oligosaccharides (sucrose, lactose, maltotriose etc.) and particularly monosaccharides (glucose, fructose, galactose) are all osmotically active molecules. From the beginning of duodenum throughout the rest of the small intestine, normal digestion sustains the osmotic pressure of the intestinal contents equal to the plasma [1], with no sudden changes in the content osmolarity. Correction of osmolarity starts in the stomach through mixing with the gastric juice, but a combination of fluid secretion and absorption of water and solutes in the small bowel maintains isotonicity of the intestinal content. As food is being slowly digested in the small bowel, new osmotically active molecules are continuously liberated from food particles, but also some of them are absorbed, so additional dilution volume by osmosis often remains limited.

The presence of hypertonic concentration of salt and/or small sugar molecules in the gut requires some intestinal fluid to dilute the gut content to isotonicity. Intact starch is not osmotically important since it is a huge molecule, but during enzymatic digestion of starch, many small molecules form and act as osmotic particles. Water follows absorption of osmotically active molecules and this process maintains isotonicity of intestinal content along the small bowel.

Due to cotransport with sodium across the mucosal membrane, many sugars and amino acids depend on the Na ^ + ^movement for their absorption from the gut. This net solute transport is important when considering the quantity of water that can be moved into the blood stream. Both glucose and galactose are in the small intestine absorbed with sodium by active cotransporters SGLT 1, so any water from drinks containing these sugars can be absorbed into the body circulation in less than two hours [1]. Fructose differs from glucose and galactose in the mechanism and speed of absorption. It is absorbed through passive carrier-mediated facilitated diffusion (GLUT 5) [13-15]. In normal adults, fructose is absorbed along the entire small bowel, so the absorption can take up to four hours. The consequence is that any water in fructose solution remains trapped until fructose is slowly absorbed.

The consequence of these differences in transport is that rapid rehydration requires solutions that contain glucose and sodium, while slower water absorption can be expected after ingestion of drinks that contain sucrose. One half water will rapidly follow the glucose absorption, but the rest is following the slow fructose absorption.

Three clinical topics are related to the dilution of intestinal content due to osmotic forces. The first is postprandial hypotension that is, at least partially, caused by osmotic water traffic into the intestinal lumen [4].

Second in diarrheal patients, hypertonic drinks with a high sugar concentration can worsen diarrhea, as they draw water out of the body and into the intestine and, thus should never be used for peroral rehydration [2], and the World Health Organization (WHO) recommended a hypotonic oral rehydration solution of salt and glucose. Hypotonic solutions made of salt and glucose polymers from rice might be even better, probably due to slowed release of glucose molecules through enzymatic digestion of polymers.

The last example is often in patients following gastric surgery, where a sudden hyperosmotic load in the small bowel can cause the "dumping syndrome", due to uncontrolled, rapid entry of hypertonic gastric content into the small intestine, under which so much water moves into the gut that significant circulatory hypovolemia and arterial hypotension result [7].

## Osmotic load in the small bowel as a trigger leading to increased colonic sodium absorption

Here proposed model is based on the idea that ingestion of hypertonic fluid transiently affects the body fluid balance due to fluid shifts needed for the intestinal content dilution. This reduction of fluid volume in circulation stimulates aldosterone secretion and increases sodium absorption in colon and reabsorption in kidneys.

**Figure 1:**
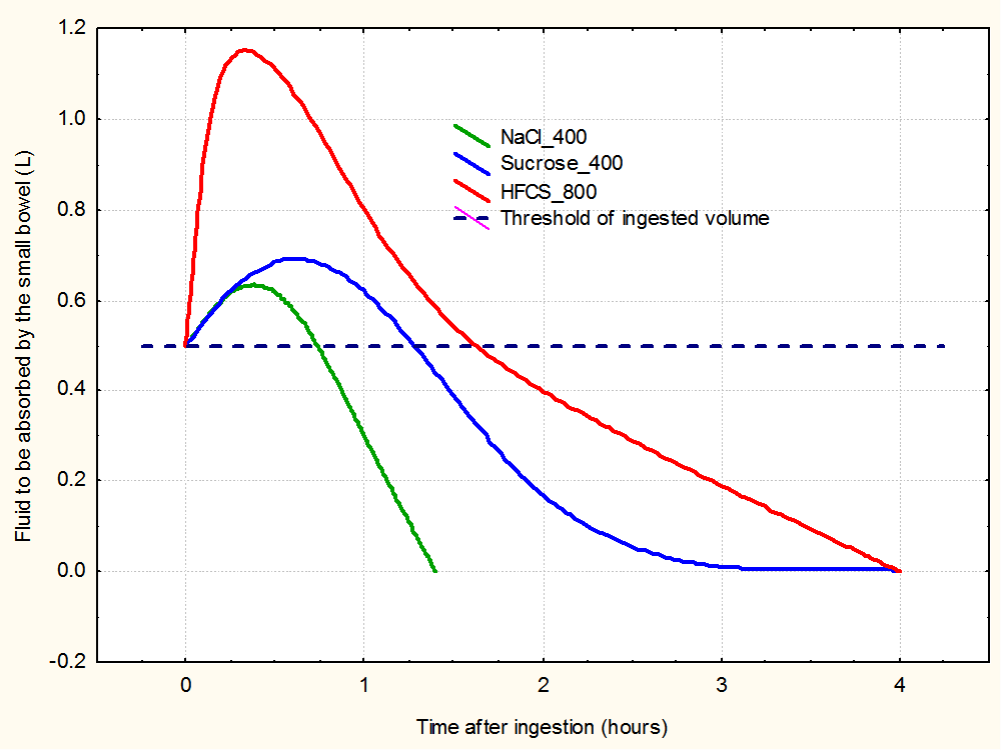
**The proposed impact of osmotic loads in the upper digestive tract on the intestinal fluid traffic (based on data from Table 1)**. Soft drinks with high fructose corn sirup (HFCS) are so hyperosmolar that they trap large volumes of intestinal fluid until their sugars are absorbed. It takes much longer for fructose [13-15]. Other liquids are moderately hypertonic and their influences on fluid traffic are much weaker.

Common fluids in our diet are listed in Table 1 with calculated dilution volumes required to achieve isotonicity. The expected volume shifts after ingestion of these fluids are shown in Fig. 1. It can be presumed that the expected total intestinal fluid volume after ingestion of a hypertonic drink is slightly less than the sum of the ingested volume and the intestinal fluid needed for dilution. If so, any hypertonic drink will initially take some water out of body into the small bowel.

**Table 1:** The proposed interplay between osmotic loads in the upper digestive tube and sodium and fluid sparing actions imposed on kidneys and large bowel by increased ADH and aldosterone secretion due to a transitory decline in the circulatory volume

Beverage	Content & overall osmolarity	Fluid cycle				Secretion	
(V = 0.5L)	(O)	Theoretic values		Fluid absorption in hours		Aldosterone	ADH
		**Initial trapped volume V**_**d**_ = V*O/300-V	**Total volume for absorption V**_**a**_** = V**_**d**_**+V**	< 2 h with	< = 4 h		
Salty commercial soup	1.3% NaCl, 400 mosm/L	~ 0.16 L	~ 0.66 L	~ 0.66 L	none	blocked by sodium absorption	increased by reduction in circulatory volume
Sucrose soft drink	13.3% sucrose, 400 mosm/L	~ 0.16 L	~ 0.66 L	~ 0.33 L with glucose	**~ 0.33 L with fructose**	weak and transitory increase in secretion due to small initially trapped volume	
HFCS soft drink	glucose 360, fructose 440, overall 800 mosm/L	~ 0.83 L	~ 1.33 L	~ 0.6 L with glucose	**~ 0.73 L with fructose**	increased secretion due to slow fructose absorption results in sodium & volume sparing in kidneys and large bowel	
WHO peroral rehydrattation solution	glucose 75, salt 170, overall 245 mosm/L	none	0.5 L	near 0.5L	almost none	rehydration without fluid trapping, hormone secretion is reduced	

Soft drinks with high fructose corn sirup (HFCS) are so hyperosmolar that they trap large volumes of intestinal fluid until their sugars are absorbed (Fig. 1.). Absorption takes longer for fructose [13-15]. Other liquids are moderately hypertonic and their influences on fluid and sodium homeostasis are much weaker. The WHO recomended solution for rehydratiopn is hypotonic so no fluid is being trapped in the small bowell.

Due to the highest initial osmolarity, the HFCS soft drink is an important osmotic challenge (Table 1 and fig. 1). Ingestion of 0.5 L of hypertonic HFCS drink with 800 mosm/L is expected to trap the total volume of more than 1 L of isotonic content in the small bowel (0.5L is ingested and the rest is dilution by the intestinal fluid). Some 45% of this enlarged fluid volume is expected to be absorbed within two hours (with glucose absorption). The rest of it would remain in the small bowel up to four hours, due to slow fructose absorption.

Water retained in the small bowel until sugar reabsorption is temporary sequestrated from the body fluid compartments. Larger trapped fluid volumes (as expected after ingestion of HFCS soft drinks) can lead to increased renin secretion and sympathetic activity, as seen in postprandial hypotension [16,17]. Renin secretion and enhanced sympathetic activity raise ADH and aldosterone secretions. These hormones retain water and sodium in kidneys and augment sodium and water absorption in the large bowel. Effects on kidneys are quick and transient, while alterations of the colonic sodium absorption depend on cell renewal [3] and take hours and days to develop or dissolve [2]. It can be expected that the current capacity of sodium absorption in the large bowel reflects the average aldosterone and ADH exposure during few previous days. An individual that on average takes several HFCS soft drinks each day can make his colon prone to sustained increased sodium absorption, despite adequate sodium ingestion and more than adequate water ingestion. This can lead to a new balance that leads to an expanded ECF compartment and increased risk of arterial hypertension. The risk can be higher in individuals with impaired fructose absorption that might further prolong water reabsorption in the small bowel [18].

Ingestion of other hypertonic liquids from Table 1 seems less important. After a salty liquid meal, some water moves into the small bowel and an increase in ADH secretion is expected, but the aldosterone response is expected to be blunted by the large sodium absorption. Mild changes in ADH exposure save some water in kidneys, but their influence on sodium absorption in the large bowel is probably weak.

A similar situation is expected with a sucrose containing soft drink. Initially, dilution does not take much water, due to mild hypertonicity of sucrose drink. Eventually, sucrose is enzymatically split in glucose and fructose and new osmotic particles take some additional water, but the amount is reduced by simultaneous reabsorption of these two sugars, particularly of glucose. So, only a limited and transient increase in ADH and aldosterone secretion can be expected.

Although highly hyperosmolar [9,12], ethanol containing beverages are deliberately avoided in Table 1. Due to rapid ethanol absorption in the upper digestive tube [19], ethanol dependent osmolarity is quickly reduced, so the initial osmotic load does not last long. Since ethanol blocks ADH secretion, only a transient aldosterone rise can be expected after taking ethanol containing drinks.

## Consequences of the proposed model

Here presented interpretations suggest that it might be important how an individual takes carbohydrates or salt. It seems that the best way to avoid the reported risk of hypertension from HFCS drinks is to dilute them with fresh water before ingestion, or to drink adequate volume of fresh water immediately after the HFCS drink. Any isotonic, or hypotonic combination of food and drinks that slowly releases both salt and sugars might help avoid risk of arterial hypertension, due to small volume of initially trapped fluid.

As of May 2010, PubMed lists some 150 papers considering HFCS in various settings. Some of them are discussing relations between HFCS ingestion and blood levels of metabolic hormones (insulin, leptin, GHrelin etc.), aiming to link HFCS to obesity and to the metabolic syndrome [20-22]. Since the link between HFCS and risk of hypertension is recently published [6], it is not suprising that data on HFCS ingestion and hypertension related endocrine or paracrine mediators are lacking in PubMed journals.

Here proposed model predicts subtle and transient changes in the circulatory volume after the ingestion of hypertonic HFCS drink. So, only a slight increase in aldosterone blood levels can be expected between one and four hours after ingestion. In other words, the risk of future hypertension would be higher in individuals that take HFCS drinks in regular intervals, along meals, in concordance with Jalal et al. [6]. A plausible extrapolation of the same model is that both regular lactulose ingestion and impaired fructose ingestion can also be linked to increased aldosterone exposure and risk of developing hypertension.

## Competing interests

The author declares that they have no competing interests.
